# Antimicrobial use and resistance in Democratic Republic of Congo: Implications and recommendations; A mini review

**DOI:** 10.1016/j.amsu.2022.104183

**Published:** 2022-07-22

**Authors:** Aymar Akilimali, Malik Olatunde Oduoye, Alain Balume, Daniel Kachunga, Pierre Luundo, Rahma Sayadi, Styves Banga, Adolphe Aganze, Guillain Ramandizi, Bonk Muhoza

**Affiliations:** Faculty of Medicine, Official University of Bukavu, Bukavu, Congo; Department of Research, Oli Health Magazine Organization, Kigali, Rwanda; College of Medical Science, Ahmadu Bello University, Zaria, Nigeria; Faculty of Medicine, Official University of Bukavu, Bukavu, Congo; Faculty of Medicine, University of Monastir, Monastir, Tunisia; Faculty of Medicine, Official University of Bukavu, Bukavu, Congo; Department of Research, Oli Health Magazine Organization, Kigali, Rwanda; Faculty of Medicine, Official University of Bukavu, Bukavu, Congo

**Keywords:** Antibiotic, Democratic republic of the Congo, Drug resistance, Infections, Public health

## Abstract

Antimicrobial resistance is a public health problem in the Democratic Republic of Congo and it occurs when pathogens (bacteria, viruses and parasites) no longer respond to antimicrobial drugs. So, their treatment becomes ineffective and complex increasing the risk the spread of infectious and opportunistic diseases and producing serious forms of these infections leading to a high number of deaths. It is important to put in place effective methods and materials for the prevention and treatment of infections resistant to antimicrobial drugs, to provide improved access to quality antimicrobials and to reduce the number of treatment failures. Increasing levels of resistance have significant economic consequences and third-line treatments become much more expensive than first- and second-line treatments. As antimicrobial resistance is a complex health problem that requires a coordinated multisector approach, it is important to intensify innovation in operational research and the development of new antimicrobial drugs. Surveillance remains a valuable tool in the fight against antimicrobial resistance in the Democratic Republic of Congo, as it helps to detect resistant infections and allows corrective decisions to be made, this surveillance guides policy recommendations and the monitoring of use and treatment and abuse of antibiotics.

Our study aimed to show the importance of establishment of national plans for the fight against antimicrobial resistance by installing laboratories and monitoring sites in order to reduce the risk and rate of deaths due to antimicrobial resistance in the Demographic republic of Congo.

## Highlights

• Antimicrobial resistance is a public health problem worldwide and in the Democratic Republic of Congo in particular.
• Currently in the Democratic Republic of Congo, there is a high rate of infections that do not respond to treatment, which increases the spread of certain diseases and the occurrence of serious forms.
• Surveillance of this resistance is valuable in the fight against antibiotic resistance because it helps to detect resistant infections and will allow the government to make decisions and put in place recommendations on the use of antibiotics.

## 1 Introduction

Antimicrobial resistance (AMR) is a major problem for the health system in the Democratic Republic of Congo (DR Congo). As AMR is a public health problem facing humanity, it is ranked among the ten major health threats according to the World Health Organization (WHO) [1]. The uncontrolled and excessive or misuse of antimicrobial drugs contributes to the resistance of pathogens against drugs. AMR occurs when pathogens (bacteria, viruses and parasites) resist the effects of drugs. For these cases, it is very difficult to treat common infections, which is a risk factor for the spread of infectious diseases [2].

The DR Congo, a country in which several tropical diseases, including bacterial infections, invaded and whose health system was destabilized during this period by the pandemic of Coronavirus 2019 (COVID-19) and a series of epidemics of the Ebola virus disease, its health system is facing AMR these days [3]. Given that the excessive use of antimicrobials is the main factor of antimicrobial resistance, multiple factors are also at the origin of this resistance in DR Congo such as the lack of access to drinking water for its population, the lack of sanitation and hygiene but also a weak measure of prevention and fight against bacterial infections contribute to amplify these threats against public health and also favor the spread of infections [4]. To our knowledge, no previous study has been carried out on the situation and implications of AMR in the region of the DR Congo. To this end, we sought to describe the current situation of AMR and analyze the implications in the clinical care of AMR. To complement this, we also describe the recommendations to take in the face of AMR.

## 2 Current situation of antimicrobial resistance in DR Congo

Nowadays, we cannot refuse that antibiotics reduce the burden of infectious diseases because without them thousands of people could have died from these infections [5]. AMR threatens the effectiveness of successful treatment of infections and constitutes a national public health problem in the DR Congo where areas are in difficulty because of the reigning war, country where there is almost no medical neutrality because of armed men who enter hospitals and demand their laws by attacking health personnel [6]. The environment is a medium facilitating the transmission of resistant bacteria and the emergence of resistant pathogens, thus wastewater, bodies of water, dust and food colonized by bacteria, are important vectors allowing bacterial transmission. between hosts through the environment [7]. In DR Congo, where the health system has been disrupted, destabilized and weakened by lack of health facilities, insufficient provision on microbial surveillance but also very high resistance to antimicrobials has been recorded (3.8). With this very high rate of resistance to antimicrobials while many infectious diseases are commonly encountered in DR Congo, there is a risk of soon observing an ineffectiveness of antibiotics in the days to come while deaths are still recorded in this region due to conditions of displacement due to armed conflicts, which in turn lead to disease [8].

Without effective antimicrobials, the success of modern medicine in treating infections, including surgery and cancer chemotherapy, would be jeopardized. Therefore, AMR is a phenomenon often observed in bacteria because of their spread which is accelerated by the irrational use of antibiotics, the use of fake drugs, bad prescription habits and poor compliance with prescribed treatments. And for the national economy of DR Congo, the cost of AMR is considerable because of deaths, the duration of illness being longer with prolonged stays in hospital and financial hardship for those affected [9,10]. The DR Congo does not have a national coordination on the monitoring and evaluation of AMR except a few microorganisms that are currently targeted, including tuberculosis and typhoid fever. Resistance to treatment for these two endemic diseases has been closely monitored for several years. DR Congo does not have sufficient medical laboratories for monitoring AMR, only a few laboratories that exist and they are often specific to hospitals and most of the tests carried out by these laboratories are only for therapeutic purposes and not surveillance of AMR [6].

## 3 Current strategies and efforts to fight antimicrobial resistance in DR Congo

AMR is a major global threat and driving up healthcare costs. The implementation of the national action plan to combat antimicrobial resistance in DR Congo by the Congolese government and its health system, will help prevent and treat infectious diseases with safe and effective drugs. In addition, nations generally divert resources in times of conflict to meet military needs rather than the health needs of the population. These unfortunate conditions provide an atmosphere for bacteria to establish traditional antibiotic resistance mechanisms as well as opportunities for the spread of emerging pathogens [6,7].

DR Congo should acquire equipment and human resources for laboratories. Laboratories that will help ensure the reliability of testing and the establishment of a quality assurance system in these laboratories will help generate adequate and reliable data on antimicrobial resistance that will guide action to address resistance to antimicrobials.

In addition to this, internal quality assurance should be carried out regularly on reagents and tests in these laboratories. They should also participate in external quality assurance carried out at the regional, national or international level. The Congolese government can also reinforce existing laboratories with modern equipment. In each of the 26 provinces of the DR Congo, they be equipped with a laboratory and be authorized as a provincial reference laboratory. This can then supervise other laboratories in the province for quality assessment. Surveillance of AMR must be done by establishing a national action plan that will bring together all the important, necessary and recommended measures required.

This will help to reduce the emergence of resistant pathogens and their spread. Moreover, the establishment of a sentinel antimicrobial resistance surveillance site, use of standardized protocols, and use of software and information systems will also assist in surveillance and monitoring of antimicrobial resistance and pathogens. to be monitored should be prioritized according to the disease burden in the country and the antibiotics selected to be tested for each pathogen should take into account the list of essential medicines used in DR Congo and treatment guidelines.

## 4 Health implications and recommendations

The fight against AMR with a moderate and effective surveillance network allows the number of pathogens to be reduced and allows more discrimination of epidemics at the earliest. It also decreases the risk of death from resistant infections. Regarding this situation, the main actions to be recommended include the establishment of surveillance, monitoring and control systems for AMR, the construction of laboratories with sufficient capacity for rapid and reliable diagnostic tests, but also the development and implementation of national action plans to combat antibiotic resistance.

AMR networks should help share information regarding antimicrobial resistance surveillance within the country and across regions and also share information regarding the risks of antibiotic resistance and the risk of irrational use of antibiotic drugs. This is also important in the case of outbreaks of pathogens of public health concern.

## 5 Conclusion

Antimicrobial Resistance is known to be a major public health in most developing countries, including the Democratic republic of Congo. Therefore, the Congolese government should establish national plans for the fight against antimicrobial resistance by installing laboratories and monitoring sites in order to reduce the risk and rate of deaths due to antimicrobial resistance in the Demographic republic of Congo.

## Ethical approval

Not applicable.

## Sources of funding

This research did not receive any specific grant from funding a commercial, or not for profit sectors.

## Author contributions

Conception: Aymar AKILIMALI, Design: Malik OLATUNDE ODUOYE, Administrative support: Aymar AKILIMALI, Supervision: Guillain RAMANDIZI, Validation: Alain BALUME and Guillain RAMANDIZI, Investigation: Styves BANGA and Daniel KACHUNGA, Resources: Daniel KACHUNGA, Visualization: Malik OLATUNDE ODUOYE and Alain BALUME, Funding acquisition: Bonk MUHOZA, Literature search: Pierre LUUNDO, Aymar AKILIMALI and Rahma SAYADI, Manuscript preparation: Aymar AKILIMALI, Malik OLATUNDE ODUOYE and Bonk MUHOZA, Manuscript editing: Aymar AKILIMALI, Pierre LUUNDO and Daniel KACHUNGA, Manuscript review: Aymar AKILIMALI, Malik OLATUNDE ODUOYE and Adolphe AGANZE, Final approval of manuscript: All Authors.

## Registration of research studies

Name of the registry: not applicable.

Unique Identifying number or registration ID: not applicable.

Hyperlink to your specific registration (must be publicly accessible and will be checked): not applicable.

## Guarantor

Aymar AKILIMALI is the guarantor of this study and accept full responsibility for the work and the conduct of the study, has access to the data and controlled the decision to publish.

## Consent

Not applicable.

## Data availability

Not applicable.

## Declaration of competing interest

The authors declare that they have no conflicts of interest.

## Abbreviation

AMR: Antimicrobial resistance
COVID-19: Coronavirus 2019
DR Congo: Democratic Republic of Congo
WHO: World Health Organization
